# The immunotherapy advancement targeting malignant blastomas in early childhood

**DOI:** 10.3389/fonc.2023.1015115

**Published:** 2023-02-16

**Authors:** Bolun Zang, Luyue Ding, Linlin Liu, Senthil Arun Kumar, Wei Liu, Chongchen Zhou, Yongtao Duan

**Affiliations:** Henan Provincial Key Laboratory of Pediatric Hematology, Children's Hospital Affiliated to Zhengzhou University, Zhengzhou University, Zhengzhou, China

**Keywords:** malignant blastomas, advanced immunotherapy, monoclonal antibodies, chimeric-antigen based receptor cell therapy, childhood

## Abstract

Malignant blastomas develop relentlessly in all functional body organs inflicting severe health ailments in younger children. Malignant blastomas exhibit diverse clinical characteristics in compliance with their emergence in functional body organs. Surprisingly, neither of these preferred treatment types (surgery, radiotherapy, and chemotherapy) showed promise or were effective in treating malignant blastomas among child patients. N ew, innovative immunotherapeutic procedures including monoclonal antibodies and chimeric-antigen based receptor (CAR) cell therapy, coupled with the clinical study of reliable therapeutic targets and immune regulatory pathways targeting malignant blastomas, have attracted the attention of clinicians recently.

## Introduction

Malignant blastomas, based on their emergence of localization in vital human organs, have been classified into various types, including neuroblastoma, retinoblastoma, hepatoblastoma, pleuropulmonary blastoma, and pancreatic blastoma. Malignant blastomas predominantly diagnosed in young children inflict severe health ailments on them. Blastomas observed in young children show diverse clinical characteristics with a profound heterogenicity (1). Immunotherapy has been unequivocally proclaimed as an effective treatment strategy to manage malignant blastomas akin to surgical therapy, chemotherapy, and radiotherapy. Nevertheless, among other proposed therapies shown above, immunotherapy has been found pertinent, efficient, and commendable with minimal side effects towards managing malignant blastomas. Moreover, unraveling novel immunotherapeutic procedures concordant with the exploration of crucial cell signaling pathways linked to malignant blastomas could benefit clinicians with an effective way of tackling severe health ailments of malignant blastomas. Children with malignant blastomas, who were exposed to immunotherapy, especially to monoclonal antibodies and chimeric-antigen based receptor (CAR) cell therapies, showed a significant improvement in their health condition with an improved lifestyle and a higher survival percentage. Also, patients showed a noticeable progress associated with a diminished recurrence of blastomas and an improved prognosis with fewer side effects than their counterparts subjected to other recommended anti-tumor therapies. This review elaborately discusses the emergence of advanced novel immunotherapies and their clinical merits in attenuating the progression of malignant blastomas and their associated detrimental health ailments on child patients.

## Neuroblastoma

Neuroblastoma principally emerges from the immature embryonic neural crest and develops into an extracranial solid tumor in the early stages of growth in younger children (2, 3). Recently, the anti-GD2-monoclonal antibody that showed higher therapeutic efficacy against neuroblastoma has been proposed in the standard regulatory treatment guidelines accepted for neuroblastoma (2, 4, 5). In contrast, this treatment remains ineffective on child patients with severe neuroblastoma wherein the survival rate was <50% even with combinatorial immunotherapy. Dinutuximab, an anti-tumor drug, showed deleterious side effects such as chronic body pain, catheter-associated infection, fever, cough, edema, and impaired liver enzyme secretions on child patients (6). Henceforth, the drug has been referred to patients with more precautions, challenging all clinicians to treat severe neuroblastoma child patients. Nevertheless, dinutuximab exposure with the chimeric anti-GD2 antibody (CH14.18) treatment showed a positive clinical outcome with a better survival rate and a significant improvement in GM-CSF, IL-2, and RA concentrations in the neuroblastoma child patients in the phase III trial (5). The study outcomes were promising and notable with positive clinical inferences compared to the clinical outcomes of 226 neuroblastoma child patients who have responded positively to induction therapy and stem cell transplantation.

**Figure 1:**
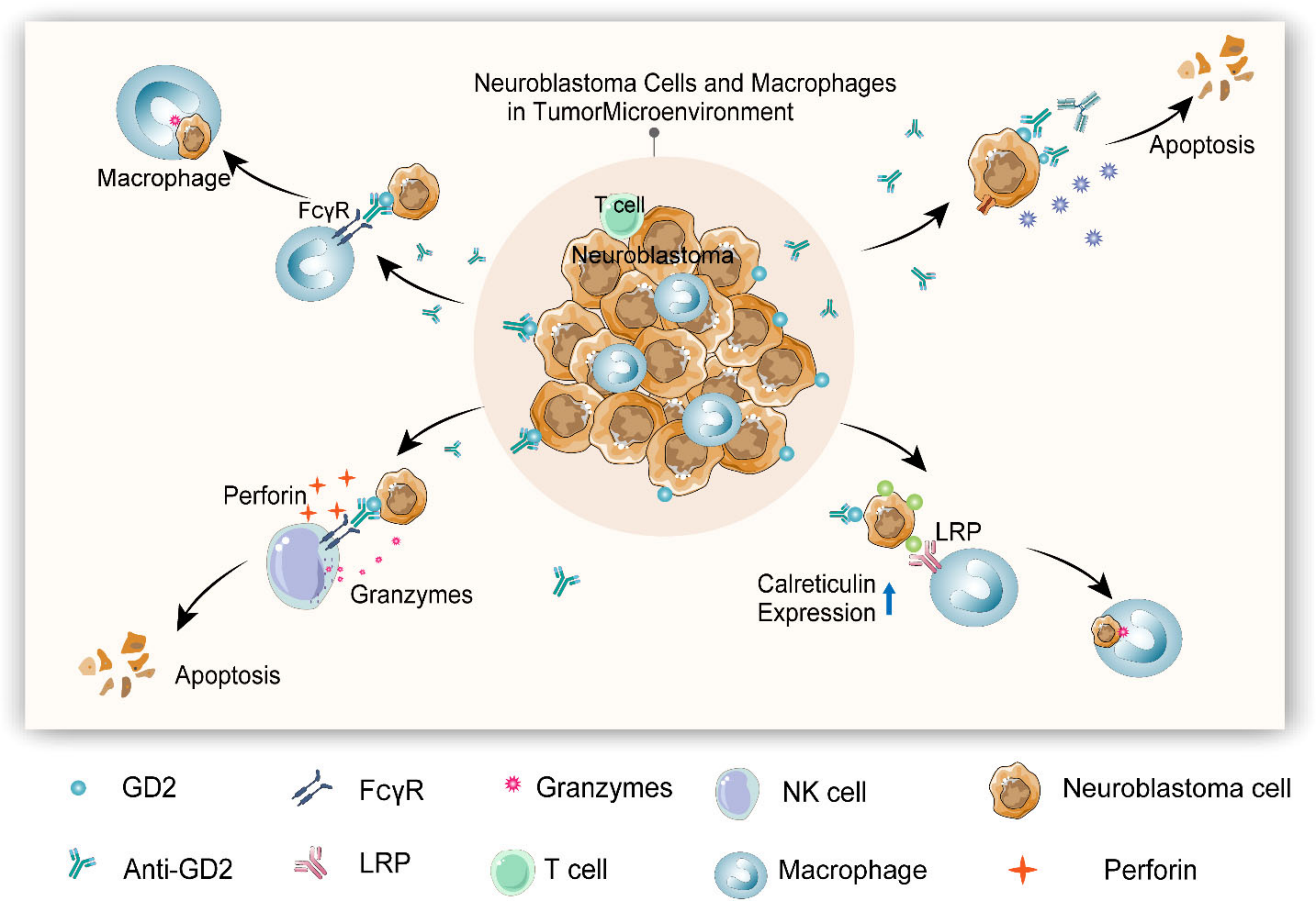
Anti-GD2-induced tumor microenvironment and cytotoxic action of neuroblastoma (1). Macrophage can be activated by anti-GD2 *via* FCγRI (CD64) and FcγRIIA (CD32), initiating phagocytosis to kill neuroblastoma cells (2). Anti-GD2 induces complement-dependent cytotoxicity (CDC) by triggering complementary activation through C1q antibody interactions to kill neuroblastoma cells (3). Anti-GD2 activates antibody-dependent cytotoxicity (ADCC) of natural killer (NK) cells *via* FcγRIIIA (CD16a), thereby releasing perforin and granulozyme to kill neuroblastoma cells (4). Anti-GD2 and other monoclonal antibodies can induce the expression of calreticulin (“eat me” molecule) on neuroblastoma cells and enhance the phagocytosis of macrophages.

In the present scenario, the anti-GD2 antibody has been clinically recommended with other treatment means to manage neuro blastoma. GD2 (CH14.18/CHO) antibody exposure as a supplementary therapy with haploidentical stem cell transplantation was effective on child patients diagnosed with stage IV recurrent neuroblastoma. Immunological inferences of this study affirmed that an exposure to anti-GD2 antibody considerably enhanced antigen-dependent cytotoxicity and complement-dependent cytotoxicity accompanied by the increased pro-inflammatory cytokines and natural killer cell responses. Moreover, the use of haploidentical stem cell transplantation in combinatorial therapy, particularly with immunotherapy, which resulted in a positive clinical outcome, can be clinically adopted to treat advanced neuroblastoma *via* spawning functional effector cells to facilitate natural killer cells or T-cell therapy (7). The mechanisms related to anti-GD2-induced tumor microenvironment and cytotoxic action of neuroblastoma are detailed in **Figure 1**.

Antigen-dependent cytotoxicity is an immunological response that attenuates tumor cells’ growth and development *via* enhanced antibody production. Dinutuximab’s antineoplastic effect has been markedly regulated by the antigen-dependent cytotoxicity stimulating antibody production. Also, dinutuximab exposure by inhibiting the crucial CD47-SIRPα marker ameliorated the neutrophil action degrading neuroblastoma development (8). This noticeable anti-neuroblastoma effect of dinutuximab is of the adrenergic phenotype, reinforcing its therapeutic proficiency against neuroblastoma.

Similarly, children with neuroblastoma exposed to anti-GD2 antibody showed a better recovery with the increased granulocyte-colony stimulating factor secretions. Research studies have assured the beneficial effect of elevated granulocyte-colony stimulating factor secretions with anti-GD2 antibody exposure providing a better clinical outcome and recovery on children with neuroblastoma (8, 9). Concomitantly, unveiling novel therapeutic targets linked to antigen-dependent cytotoxicity is crucial to ameliorate its therapeutic efficacy in targeting neuroblastoma.

However, studies have shown that subcutaneous administration of interleukin-2 (10, 11) while treating with intravenous dinutuximab triggered adverse symptoms of neuroblastoma with unprecedented inflammatory responses (9). Clinical trial validation of this combinatorial therapy affirming its appropriate dosage level is crucial before experimenting with this on children with neuroblastoma. An early review showed that anti-GD2 antibody (CH14.18) is ineffective in reversing the adverse health ailments and associated mortality in child patients (12). Yu et al.’s (13) clinical follow-up showed dinutuximab ameliorating the survival rate but eventually turned out to be inefficient in the later stages of development in children with neuroblastoma. Naxitamab, a newly developed anti-GD2 antibody, has been accredited by the US Food and Drug Administration (FDA) to treat refractory and/or recurrent neuroblastoma augmented with GM-CSF (14). Naxitamab treatment was found to be effective, with positive clinical outcomes on neuroblastoma child patients identified only with bone or bone marrow ailments in severe refractory/relapsed conditions. This clinical phase II trial was conducted in the recent international center that constitutes 48 normal control subjects and 36 children with neuroblastoma under the above clinical conditions. Nearly 58% of children with neuroblastoma (21 of 36) responded well to naxitamab therapy, 44% (16 of 36) of whom showed a complete recovery. Another 16% showed only a partial recovery with naxitamab therapy. The patients’ clinical responses were examined in a time interval of 25 weeks. Common side effects associated with infusion, including pain, were unanimously witnessed among all study patients. Simultaneously, 13 patients showed detrimental side effects with naxitamab treatment, of whom 4 patients were hypotensive, 4 patients had anaphylaxis, and the remaining 5 patients were each diagnosed with fatigue, fever, laryngeal edema, respiratory depression, and urticaria. Furthermore, four patients were expelled from the clinical trial after they developed severe adverse reactions to naxitamab. Surprisingly, there were no mortality recorded at any level of the clinical trials. Also, in detail, clinical trials have to be conducted meticulously to affirm the therapeutic efficacy of naxitamab against neuroblastoma (3). Clinical trials in progress deployed Naxitamab treatment for neuroblastoma are detailed in **Table 1**.

**Table 1:** Clinical trials in progress used naxitamab treatment for neuroblastoma.

ClinicalTrials.gov Identifier	Type of study	The number of estimated enrollment	Estimated study completion date	Current primary outcome measures	Brief title	Drugs and methods
NCT05489887	Phase 2	76 participants	September 2033	Number of participants with a VGPR (+) rate (VGPR +CR rate) [Time Frame: Month 12]	Naxitamab Added to Induction for Newly Diagnosed High-Risk Neuroblastoma	Naxitamab + GM-CSF + Isotretinoin
NCT03363373	Phase 2	95 participants	November 2027	Response rate during Naxitamab treatment [Time Frame: 101 weeks]	Naxitamab for High-Risk Neuroblastoma Patients with Primary Refractory Disease or Incomplete Response to Salvage Treatment in Bone and/or Bone Marrow	Dinutuximab with Chemotherapy

VGPR, very good partial response; CR, complete response; GM-CSF, granulocyte-macrophage colony-stimulating factor.

**Figure 2:**
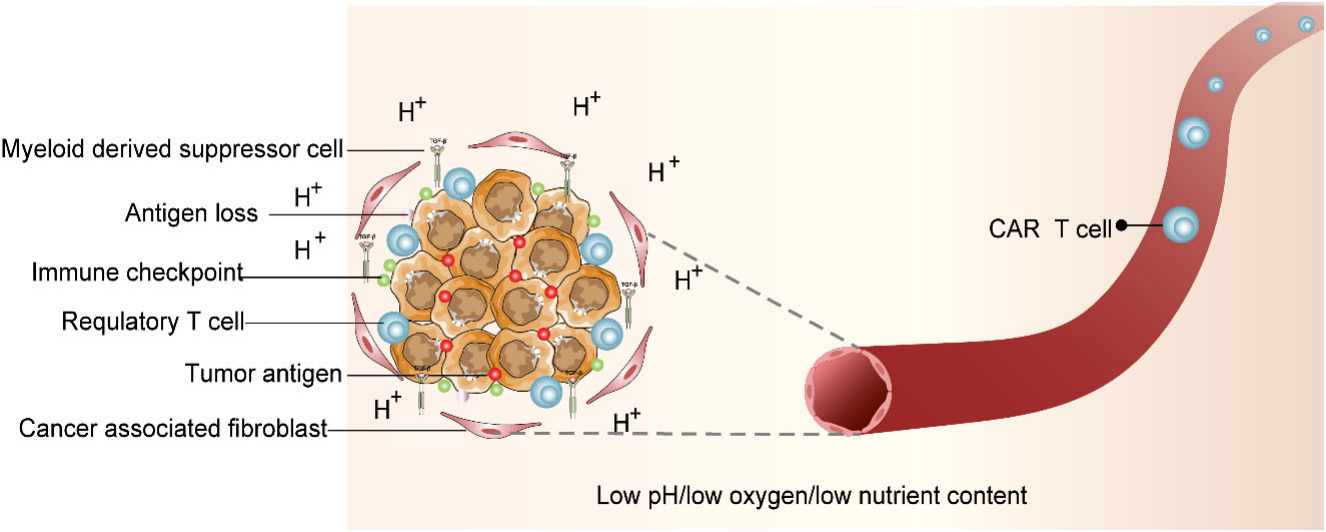
Immunosuppressive tumor microenvironment (TME). CAR T-cell therapy is entangled with difficulties. TME that deteriorates CAR T-cell survival is characterized by acidic pH, oxidative stress, nutritional depletion, and hypoxia. In addition, associated antigen loss, inhibitory cytokines and regulatory T cells (Tregs), myeloid-derived suppressor cells (MDSCs), tumor-associated macrophages (TAMs), and cancer-associated fibroblasts (CAFs) were factors that influence CAR T-cell activity.

We believe that any T cell has an extremely sensitive and specific T-cell receptor (TCR) that can detect abnormal signals of the body and trigger a cascade of immune responses when the abnormal peptide chain is identified as the pathogen. Chimeric antigen receptor (CAR) T -cell therapy has become the most popular therapeutic method in cancer individualized immunotherapy. CAR T-cell therapy principally employs gene editing that facilitates T cells to target tumor cell-specific antigens through gene modification intended to kill target-specific tumor cells. For the CAR T-cell therapy, the T cells from patients’ site-specific tumor tissues will be derived and then injected back into the patients’ bodies after *in vitro* gene editing and amplification to produce pronounced anti-tumor effects (15). CAR T-cell therapy has shown promising clinical outcomes in patients diagnosed with acute lymphoblastic leukemia (16). However, due to side effects, antigen escape, and immune suppressive tumor microenvironment (TME), the CAR T-cell therapeutic effect on solid tumors is not satisfactory. Although CAR T-cell therapy has found many alternative targets in the treatment of solid tumors, most of them show limited anti-tumor effects in clinical trials (15, 17, 18). The application of CAR T-cell therapy in attenuating solid tumors demands more clinical studies. The clinical challenges in CAR T-cell therapy are illustrated in **Figure 2**.

Studies have revealed that CAR T-cell therapy did not show any noticeable therapeutic effect on neuroblastoma patients (19). Research studies evaluating the therapeutic proficiency of CAR T-cell therapy against neuroblastoma in young children have been widely performed. SynNotch-based GD2-B7H3 CAR T-cell therapy is a distinct immunotherapy developed with higher specificity and therapeutic proficiency (to target neuroblastoma) that employs gated CAR T cells, which are more promising than conventional CAR T cells in terms of anti-tumor effect. This inference is made upon its supreme metabolic adaptation and minimal depletion levels. To ameliorate the therapeutic effect of CAR T cells on neuroblastoma, indoleamine-pyrrole 2, 3-dioxygenase 1 (IDO1), which attenuates the anti-tumor responses of anti-GD2 CAR T cells and natural killer cells, has been subjected to clinical phase I/II trials, offering a better clinical outcome in treating neuroblastoma (20). Clinical trials in progress employed CAR-T treatment for neuroblastoma are detailed in **Table 2**. Collectively, CAR T-cell therapy was effective in treating neuroblastoma.

**Table 2:** Clinical trials in progress employed CAR-T treatment for neuroblastoma.

ClinicalTrials.gov Identifier	Type of study	The number of estimated enrollment	Estimated study completion date	Current primary outcome measures	Brief title	Drugs and methods
NCT04637503	Phase 1 Phase 2	100 participants	31 December 2023	Number of patients with adverse events [Time Frame: 3 years]	4SCAR-T Therapy Targeting GD2, PSMA, and CD276 for Treating Neuroblastoma	GD2, PSMA, and CD276 CAR T cells
NCT03373097	Phase 1 Phase 2	42 participants	December 2027	Phase I —Identification of the DLT [Time Frame: 4 weeks after T-cell infusion]	Anti-GD2 CAR T Cells in Pediatric Patients Affected by High Risk and/or Relapsed/Refractory Neuroblastoma or Other GD2-Positive Solid Tumors	GD2-CART01
NCT02311621	Phase 1	65 participants	November 2038	DLT [Time Frame: 28 days]	ENCIT-01	Patient-derived CD171-specific CAR T cells expressing EGFRt (2nd-generation T cells/3rd-generation T cells/long spacer 2nd-generation T cells)
NCT03721068	Phase 1	18 participants	19 September 2038	Number of participants with adverse events as a measure of safety and tolerability of iC9.GD2.CAR.IL-15 T cells administered to pediatric subjects with relapsed or refractory neuroblastoma or relapsed/refractory osteosarcoma [Time Frame: 4 weeks]	Study of CAR T Cells Targeting the GD2 with IL-15+iCaspase9 for Relapsed/Refractory Neuroblastoma or Relapsed/Refractory Osteosarcoma	iC9.GD2.CAR.IL-15 T cells, Cyclophosphamide, and Fludarabine
NCT01822652	Phase 1	14 participants	October 2030	Dose-limiting toxicities at 6 weeks post T-cell infusion [Time Frame: 6 weeks after infusion of the last dose of iC9-GD2 T cells to all patients on the study]	3rd-Generation GD-2 Chimeric Antigen Receptor and iCaspase Suicide Safety Switch, Neuroblastoma, GRAIN	iC9-GD2 T Cells —frozen/iC9-GD2 T Cells —fresh/iC9-GD2 T cells, Cytoxan, Fludara, Keytruda
NCT03294954	Phase 1	24 participants	10 August 2034	MTD of autologous NKTs expressing a 2nd-generation GD2-specific chimeric antigen receptor administered to patients with relapsed or refractory neuroblastoma. [Time Frame: 28 days]	GD2-Specific CAR and Interleukin-15 Expressing Autologous NKT Cells to Treat Children with Neuroblastoma	GINAKIT Cells, Cytoxan, Fludara
NCT00085930	Phase 1	18 participants	December 2021	Evaluate the safety of escalating doses of 14g2a.zeta chimeric receptor transduced autologous EBV-CTL and 14g2a.zeta transduced autologous peripheral blood T cells [Time Frame: 15 years]	Blood T Cells and EBV-Specific CTLs Expressing GD-2 Specific Chimeric T-Cell Receptors to Neuroblastoma Patients	EBV-specific CTLs
NCT01953900	Phase 1	26 participants	31 October 2034	Number of patients with DLT [Time Frame: 6 weeks]	iC9-GD2-CAR-VZV-CTLs/Refractory or Metastatic GD2-positive Sarcoma and Neuroblastoma	GD2 T cells, VZV vaccine, Fludara, Cytoxan
NCT04864821	Early Phase 1	24 participants	14 May 2023	AE [Time Frame: 2 years after treatment] ORR [Time Frame: 12 weeks after treatment] Cmax [Time Frame: 2 years after treatment]	Clinical Study of CD276 Targeted Autologous Chimeric Antigen Receptor T-Cell Infusion in Patients with CD276 Positive Advanced Solid Tumor	Targeting CD276 CAR T cells
NCT04539366	Phase 1	67 participants	1 August 2024	Feasibility of producing GD2CART cells [Time Frame: up to 28 days after cell infusion] Incidence of AEs [Time Frame: up to 15 years] MTD [Time Frame: up to day 28 days after cell infusion] Best response to GD2CART cells [Time Frame: up to day 28 days after cell infusion]	Testing a New Immune Cell Therapy, GD2CART, in Children, Adolescents, and Young Adults with Relapsed/Refractory Osteosarcoma and Neuroblastoma, The GD2-CAR PERSIST Trial	Cyclophosphamide, Fludarabine Phosphate, GD2-CAR-expressing Autologous T lymphocytes
NCT04897321	Phase 1	32 participants	1 March 2027	Safety of B7-H3-CAR T cells [Time Frame: 6 weeks after B7-H3-CAR T-cell infusion]	B7-H3-Specific Chimeric Antigen Receptor Autologous T-Cell Therapy for Pediatric Patients with Solid Tumors (3CAR)	Fludara, Cytoxan, MESNA, B7-H3 CAR T cells
NCT03618381	Phase 1	36 participants	June 2038	MTD and DLT, and describe the full toxicity profile of the two CAR T-cell products [Time Frame: 28 days] The number of successfully manufactured EGFR806 and EGFR806xCD19 CAR T-cell products will be assessed [Time Frame: 28 days] Establish the safety, defined by adverse events, of EGFR806-specific CAR T-cell infusion s (Arm A), and of dual transduced EGFR806xCD19 CAR T-cell infusion s (Arm B) [Time Frame: 28 days]	EGFR806 CAR T-Cell Immunotherapy for Recurrent/Refractory Solid Tumors in Children and Young Adults	Second-generation 4-1BBζ EGFR806-EGFRt, second -generation 4-1BBζ EGFR806-EGFRt, and a second-generation 4 1BBζ CD19-Her2tG
NCT03635632	Phase 1	94 participants	December 2037	MTD of C7R-GD2.CART Cells [Time Frame: 4 weeks post T-cell infusion]	C7R-GD2.CART Cells for Patients with Relapsed or Refractory Neuroblastoma and Other GD2 Positive Cancers (GAIL-N)	C7R-GD2.CART cells, Cytoxan, Fludara
NCT04483778	Phase 1	68 participants	December 2040	Assess the safety and tolerability of cellular immunotherapy utilizing *ex vivo* expanded autologous T cells genetically modified to express B7H3-specific CAR (Arm A) [Time Frame: 28 days] Assess the safety and tolerability of cellular immunotherapy utilizing *ex vivo* expanded autologous T cells genetically modified to express a bispecific B7H3xCD19 CAR (Arm B) [Time Frame: 28 days] Determine the MTD of B7H3-specific CAR (Arm A) [Time Frame: 28 days] Determine the MTD of bispecific B7H3xCD19 CAR (Arm B) [Time Frame: 28 days] Assess the DLTs and describe the full toxicity profile for each study arm [Time Frame: 28 days] Assess the feasibility of manufacturing B7H3-specific CARs from patient-derived lymphocytes [Time Frame: 28 days] Assess the feasibility of manufacturing B7H3xCD19 bispecific CARs from patient-derived lymphocytes [Time Frame: 28 days]	B7H3 CAR T-Cell Immunotherapy for Recurrent/Refractory Solid Tumors in Children and Young Adults	Second-generation 4-1BBζ B7H3-EGFRt-DHFR, second -generation 4-1BBζ B7H3-EGFRt-DHFR (selected), and a second-generation 4-1BBζ CD19-Her2tG
NCT05562024	Phase 1	24 participants	18 February 2039	MTD [Time Frame: about 3 years] RP2D [Time Frame: about 3 years] Assessment of the safety after B7-H3-targeted CAR T-cell infusion (Safety) [Time Frame: about 3 years]	TAA06 Injection in the Treatment of Patients with B7-H3-positive Relapsed/Refractory Neuroblastoma	T-cell injection targeting FLT3 chimeric antigen receptor

CAR T cell, chimeric antigen receptor T cell; DLT, dose-limiting toxicity; ENCIT, engineered neuroblastoma cellular immunotherapy; EBV- CTL, EBV-specific cytotoxic T lymphocytes; AE, adverse event; ORR, objective remission rate; Cmax, the highest concentration of CAR T cells in peripheral blood after infusion; MTD, maximum tolerated dose; GD2CART, GD2-CAR-expressing autologous T lymphocytes; RP2D, recommended phase 2 dose.

## Retinoblastoma

Retinoblastoma (RB) is a commonly diagnosed systemic intraocular tumor in child patients and is found to be detrimental and aggressive, mainly infiltrating retinal orbit and intracranial tissues. RB is inflicted by the bi-allelic loss regulating RB gene function. Surgical resection, radiotherapy, and chemotherapy are the available treatment options recommended to manage RB in children. Among these treatment choices, chemotherapy was perceived to be effective compared to the other recommended options, greatly attenuating tumor metastasis and its progression but still considered ineffective on refractory/severely ill children with RB (21, 22).

Recently, immunotherapy as a mode of treatment for children with RB diagnosed with severe retinal ailments has attracted the attention of clinicians.

**Figure 3:**
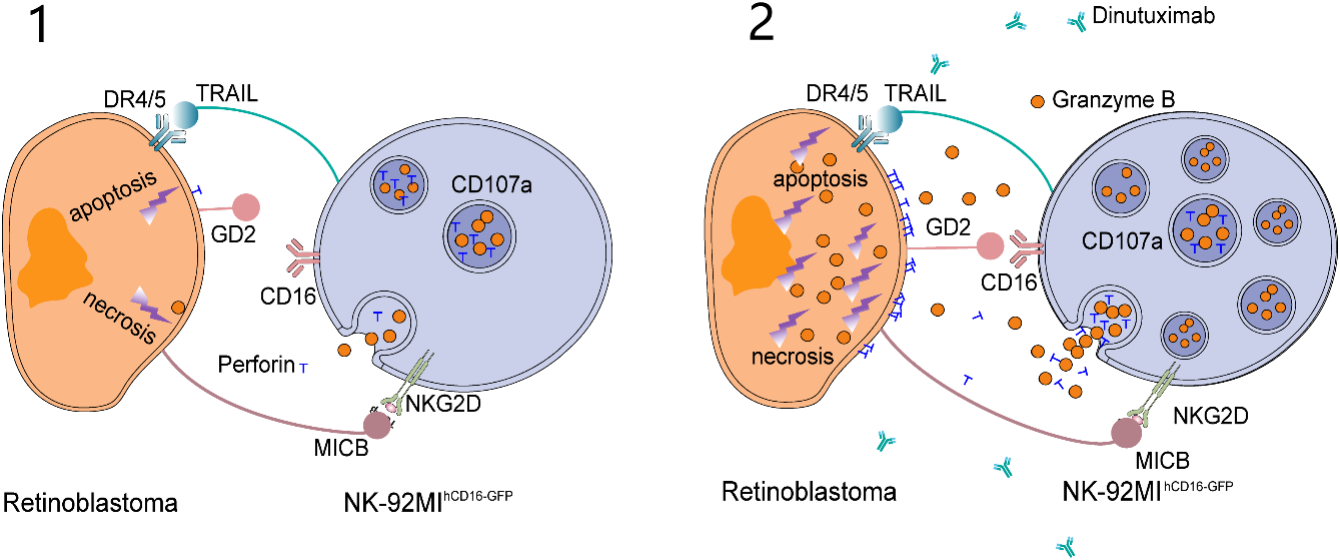
Wang et al. proposed the killing model of NK-92MI^hCD16-GFP^ cells and dinutuximab to retinoblastoma cells. (1) NK-92MI^hCD16-GFP^ cells can induce weak apoptosis and necrosis of retinoblastoma cells through intrinsic active receptors on NK-92MI^hCD16-GFP^ cells and minimum perforin and granzyme B released by NK-92MI^hCD16-GFP^ cells. (2) As a bridge between GD2 on retinoblastoma cells and CD16 on NK-92MI^hCD16-GFP^ cells, dinutuximab triggers a strong antibody-dependent cytotoxicity (ADCC) effect and leads to massive death of retinoblastoma cells. At the same time, CD107a protects NK-92MI^hCD16-GFP^ cells from cytotoxic granules.

Dinutuximab exposure to CD16-expressing NK-92MI (NK-92MI^hCD16-GFP^) cells and RB cells markedly triggered apoptosis with elevated lactic dehydrogenase (LDH) levels. Concomitantly, further exposure of RB cells in the same experimental setup involving dinutuximab with NK-92MI^hCD16-GFP^ cells enhanced perforin granase B release and CD107a expression (23). Overall, dinutuximab administration with NK-92MI^hCD16-GFP^ cells exhibited its notable anti-tumor effect, especially RB *via* anti- bot-dependent cell-mediated cytotoxicity. The mechanism by which the NK-92MI^hCD16-GFP^ cells exhibits their anti-tumor effect against RB is illustrated in two levels in **Figure 3**.

An enhanced B7H3 expression positively correlated with tumor metastasis and prognostic malignancy can be a noticeable therapeutic target in managing RB and its associated retinal ailments. Also, clinical findings affirmed increased B7H3 expression in RB cells compared to normal retinal cells, emphasizing it as a promising target of RB management (21).

Earlier clinical studies have shown CAR T-cell therapy to be effective against RB. U pon co- culturing with GD2-antigen and GD2-CAR T cells, nearly 80% of RB cells died in 24 h, and approximately 100% cell mortality was achieved in 3 days (24).

In an *in vivo* mouse model, exposure of GD2-CAR T cells to IL-15, which specifically releases IL-15 in chitosan-PEG injectable hydrogel accompanied by RB tumor cell implantation, controlled tumor cell proliferation in approximately 60% of mice in 70 days (25). Alternatively, mice administered with GD2-CAR T cells in chitosan-PEG injectable hydrogel showed RB development. An *in vitro* study showed that RB induced CD-171- and GD2-specific CAR T-cell activation, undermining tumor development, irrespective of the length of the extracellular spacer and costimulatory domains. Also, the study revealed CD-171 and GD2 as notable targets of CAR T cells, warranting an in- depth clinical validation study using an *in vivo* model (26).

## Glioma

Glioma, a tumor predominantly seen in the central nervous system (CNS) of adults, can also affect younger children causing similar health ailments. Glioblastoma is a notable glioma tumor exhibiting a higher degree of malignancy with a diverse heterogenicity in its clinical prevalence. With its notable heterogenicity, glioblastoma remains immune to chemoradiotherapy. Even with surgical therapy, glioblastoma attenuation is not abundantly feasible due to its higher malignancy. Above all, children with glioblastoma show a poor prognosis augmented with a diminished mean survival rate. Compared with these recommended treatments, immunotherapy was identified to be unique and promising, concentrating on the regulation of human host–immune system against glioblastoma emergence on the affected children. Compared with other treatments, immunotherapy may be precise, appropriate, and sustainable (27). Clinicians have focused on immunotherapy recent ly to improve the prognosis of glioblastoma in child patients. However, the use of checkpoint inhibitor monotherapy against glioma was discouraging to date, warranting further clinical trials.

Glioblastoma without tumor -specific antigen gene-protein expression and, thus, resistant to immunotherapy is labeled “cold tumor” (28). Alternatively, a tumor that is susceptible to immunotherapy is labeled “hot tumor”. Also, glioblastoma can undermine the host–immune responses in retaliation by permitting lower tumor -infiltrating lymphocytes compared with the other malignant blastomas (29). At present, immunotherapy for glioblastoma includes tumor vaccine, CAR T-cell therapy, tumor site-specific therapy using tumor-infiltrating lymphocytes, T-cell receptor therapy, and other immunosuppressants.

**Figure 4:**
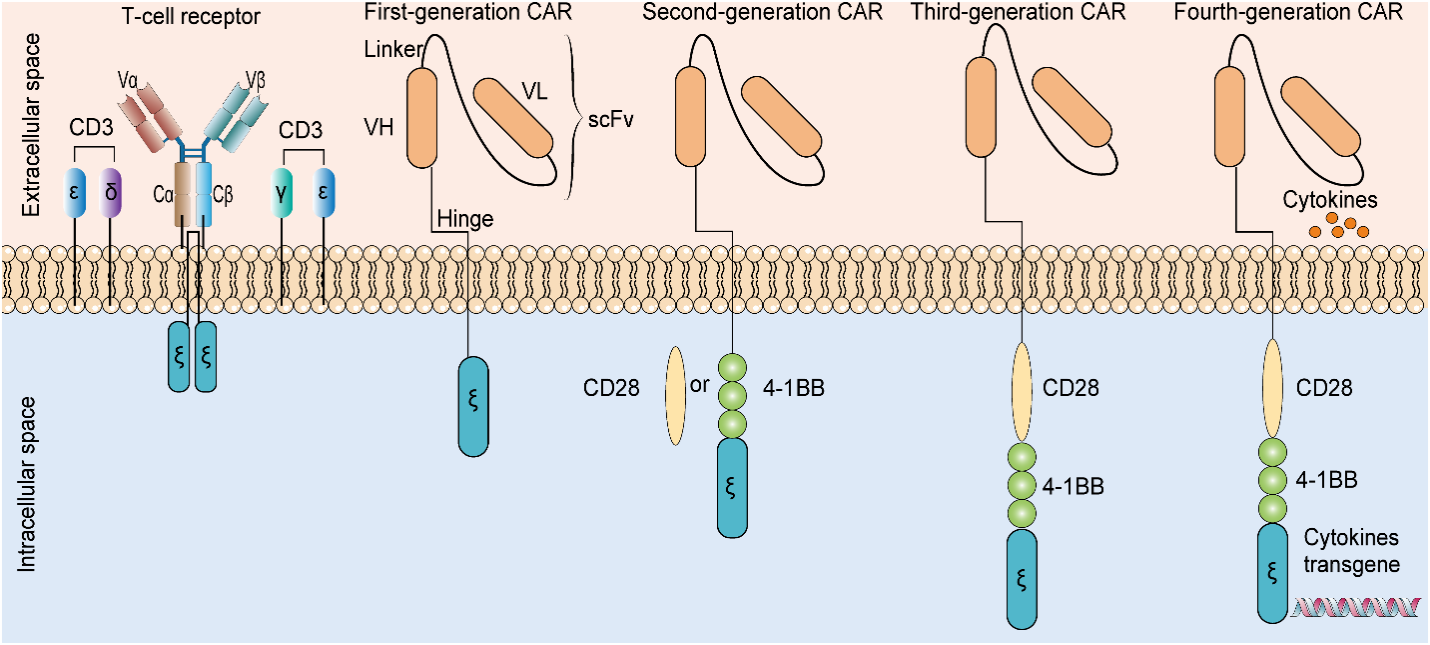
Evolution from T-cell receptor mimetics to the fourth-generation chimeric antigen receptor. The presence of additional cytokine transgenes, costimulatory domains, and T-cell signal domains in four generations of CAR are different. CAR: chimeric antigen receptor. scFV: single-chain variable fragment. VH: variable heavy chain. VL: variable light chain.

The well-studied therapeutic targets for CAR T-cell therapy in glioma include IL13Rα2, EGFRvIII, HER2, and GD2. Clinical trials conducted on these biomarkers (as tumor targets) for CAR T-cell therapy showed promising results in treating glioblastoma despite the small number of studies (15). Evolution from T-cell receptor mimetics to the fourth-generation chimeric antigen receptor are detailed in **Figure 4**. Moreover, patients subjected to therapeutic treatment, particularly single therapeutic targets, become vulnerable to relapse/drug resistance. Therefore, the use of a variety of antigens or immunosuppressive cytokines is recommended to manage tumor development. Afterwards, it is mandatory to widely explore the target antigen library to manage glioma. EphA2, B7H3, and NKG2DL have been unveiled as the new therapeutic targets for CAR T-cell therapy against glioma, and relevant clinical trials are ongoing (15). Clinical trials in progress employing CAR-T treatment for tackling glioma are detailed in **Table 3**.

**Table 3:** Clinical trials in progress employing CAR T treatment for managing glioma.

ClinicalTrials.gov Identifier	Type of study	The number of estimated enrollment	Estimated study completion date	Current primary outcome measures	Brief title	Drugs and methods
NCT03423992	Phase 1	100 participants	30 January 2023	Adverse events attributed to the administration of the chimeric antigen receptor T cells [Time Frame: 1 year]	Personalized Chimeric Antigen Receptor T-Cell Immunotherapy for Patients with Recurrent Malignant Gliomas	EGFRvIII, IL13Rα2, Her-2, CD133, EphA2, GD2 CAR T cells
NCT04185038	Phase 1	90 participants	May 2041	Establish the safety, defined by the adverse events, of B7H3-specific CAR T-cell infusion s delivered by a CNS catheter into the tumor resection cavity or ventricular system [Time Frame: up to 7 months] Establish the feasibility, defined by the ability to produce and administer CAR T-cell product, of B7H3-specific CAR T-cell product infusions delivered by a CNS catheter into the tumor resection cavity or ventricular system [Time Frame: 28 days]	Study of B7-H3-Specific CAR T-Cell Locoregional Immunotherapy for Diffuse Intrinsic Pontine Glioma/Diffuse Midline Glioma and Recurrent or Refractory Pediatric Central Nervous System Tumors	SCRI-CARB7H3(s); B7H3-specific CAR T cells
NCT05474378	Phase 1	39 participants	August 2025	Number of successful manufacturing product (B7-H3CART) that met minimum assigned dose level range [Time Frame: 5 years] MTD or recommended phase 2 dose (RP2D) [Time Frame: 5 years]	B7-H3 Chimeric Antigen Receptor T Cells (B7-H3CART) in Recurrent Glioblastoma Multiforme	B7-H3CART
NCT05366179	Phase 1	36 participants	May 2030	Number of participants with adverse event [Time Frame: up to 10 weeks] Cytokine Release Syndrome [Time Frame: up to 10 weeks] Neurotoxicity [Time Frame: up to 10 weeks]	Autologous CAR T Cells Targeting B7-H3 in Recurrent or Refractory GBM CAR.B7-H3Tc	CAR.B7-H3T cells infusion
NCT05241392	Phase 1	30 participants	31 December 2024	Incidence of DLT [Time Frame: 3 months post CAR T-cell infusion] Safety: Incidence and severity of adverse events [Time Frame: 3 months post CAR T-cell infusion]	Safety and Efficacy Study of Anti-B7-H3 CAR T-Cell Therapy for Recurrent Glioblastoma	B7-H3-targeting CAR T cells
NCT04077866	Phase 1Phase 2	40 participants	1 July 2024	OS [Time Frame: 2 years, up to 15 years if necessary]	B7-H3 CAR-T for Recurrent or Refractory Glioblastoma	B7-H3 CAR-T Temozolomide
NCT04550663	Phase 1	10 participants	25 March 2023	MTD [Time Frame: 90 days post-infusion] The occurrence of AEs and SAEs during the study treatment [Time Frame: 0 to 28 days post-infusion]	NKG2D CAR-T (KD-025) in the Treatment of Relapsed or Refractory NKG2DL+ Tumors	KD-025 CAR T cells
NCT05131763	Phase 1	3 participants	1 December 2023	OS [Time Frame: 2 years post-infusion] PFS [Time Frame: 2 years post-infusion] Partial response rate [Time Frame: through study completion, an average of 3 months]	NKG2D-based CAR T-cell Immunotherapy for Patients with r/r NKG2DL+ Solid Tumors	NKG2D-based CAR T cells
NCT05540873	Phase 1	18 participants	30 April 2024	DLT [Time Frame: 28 days after IP administration] MTD [Time Frame: 28 days after IP administration] RP2D [Time Frame: 28 days after IP administration]	A Clinical Study of IL13Rα2 Targeted CAR-T in Patients with Malignant Glioma (MAGIC-I)	YYB-103
NCT04196413	Phase 1	54 participants	31 July 2042	Radiographic Response Rate, OS, PFS [Time Frame: Day 28, 3 months, 6 months, 9 months, 12 months, and 24 months post-CAR T-cell infusion] PPS [Time Frame: Day 28, 3 months, 6 months, 9 months, 12 months, and 24 months post-CAR T-cell infusion] Measure resolution of toxicity [Time Frame: 72 h of administration of AP1903]	GD2 CAR T Cells in Diffuse Intrinsic Pontine Gliomas (DIPG) and Spinal Diffuse Midline Glioma (DMG)	GD2 CAR T cells Fludarabine Cyclophosphamide
NCT05063682	Phase 1	10 participants	October 2023	Incidence of adverse events [Time Frame: up to 10 years] OS [Time Frame: 12 months]	The Efficacy and Safety of Brain-targeting Immune Cells (EGFRvIII-CAR T Cells) in Treating Patients with Leptomeningeal Disease from Glioblastoma. Administering Patients EGFRvIII -CAR T Cells May Help to Recognize and Destroy Brain Tumor Cells in Patients	EGFRvIII-specific hinge-optimized CD3 ζ-stimulatory/41BB-co-stimulatory CAR T cells
NCT05544526	Phase 1	12 participants	December 2039	Toxicity evaluated by the incidence of grade 3–5 toxicity causally related to the ATIMP [Time Frame: 28 days] Feasibility of manufacturing GD2 CAR T cells evaluated by the number of therapeutic products generated [Time Frame: 28 days]	CAR T Cells to Target GD2 for DMG	GD2 CAR T cells
NCT04214392	Phase 1	36 participants	6 February 2023	DLT [Time Frame: 28 days]	Chimeric Antigen Receptor (CAR) T Cells with a Chlorotoxin Tumor-Targeting Domain for the Treatment of MMP2+ Recurrent or Progressive Glioblastoma	Chlorotoxin (EQ)-CD28-CD3zeta-CD19t-expressing CAR T lymphocytes (*via* ICT delivery) Chlorotoxin (EQ)-CD28-CD3zeta-CD19t-expressing CAR T lymphocytes (*via* ICT/ICV dual delivery)
NCT05298995	Phase 1	54 participants	May 2037	Safety and definition of the MTD/RD [Time Frame: 4 weeks after CAR T-cell infusion]	GD2-CAR T Cells for Pediatric Brain Tumors	GD2-CART01 (iC9-GD2-CAR T cells)
NCT04099797	Phase 1	34 participants	February 2038	DLT rate [Time Frame: 4 weeks post T-cell infusion]	C7R-GD2.CAR T Cells for Patients with GD2-expressing Brain Tumors (GAIL-B)	(C7R)-GD2.CART cells Cyclophosphamide Fludarabine
NCT03500991	Phase 1	48 participants	26 July 2039	Establish the safety, defined by the adverse events, of HER2-specific CAR T-cell infusion s delivered by a CNS catheter into the tumor resection cavity or ventricular system [Time Frame: up to 6 months] Establish the feasibility, defined by the ability to produce and administer CAR T-cell product, of HER2-specific CAR T-cell product infusions delivered by a CNS catheter into the tumor resection cavity or ventricular system [Time Frame: 28 days]	HER2-specific CAR T-Cell Locoregional Immunotherapy for HER2-Positive Recurrent/Refractory Pediatric CNS Tumors	ER2-specific CAR T cells
NCT03638167	Phase 1	36 participants	March 2040	Establish the safety, defined by the adverse events, of EGFR806-specific CAR T-cell infusion s delivered by a CNS catheter into the tumor resection cavity or ventricular system [Time Frame: up to 6 months] Establish the feasibility, defined by the ability to produce and administer CAR T cell product, of EGFR806-specific CAR T-cell product infusions delivered by a CNS catheter into the tumor resection cavity or ventricular system [Time Frame: 28 days]	EGFR806-Specific CAR T-Cell Locoregional Immunotherapy for EGFR-Positive Recurrent or Refractory Pediatric CNS Tumors	GFR806-specific CAR T cells
NCT03941626	Phase 1Phase 2	50 participants	1 December 2021	Number of participants with adverse events evaluated with NCI CTC AE, version 4.0 [Time Frame: 48 months]	Autologous CAR-T/TCR-T Cell Immunotherapy for Solid Malignancies	CAR-T/TCR-T cell immunotherapy
NCT04903080	Phase 1	50 participants	3 July 2040	Number of subjects with DLT in Phase I arm; Number of subjects with DLT in surgical arm [Time Frame: up to 42 days following the first CAR T-cell infusion] Percentage of subjects whose treatment delivery meets feasibility criteria [Time Frame: approximately 3 months after enrollment for treatment]	HER2-Specific Chimeric Antigen Receptor (CAR) T Cells for Children with Ependymoma	HER2-Specific CAR T Cell
NCT02208362	Phase 1	82 participants	18 December 2022	Incidence of grade 3 toxicity [Time Frame: up to 15 years] Incidence of DLT, graded using NCI CTC AE version 4.0 [Time Frame: up to 1 week following the last course (not including optional courses 4–6)] Incidence of toxicities, graded using NCI CTC AE version 4.0 as well as the modified neurological grading system [Time Frame: up to 15 years]	Genetically Modified T Cells in Treating Patients with Recurrent or Refractory Malignant Glioma	IL13Ralpha2-specific Hinge-optimized 4-1BB-co-stimulatory CAR/Truncated CD19-expressing Autologous TN/MEM Cells IL13Ralpha2-specific Hinge-optimized 41BB-co-stimulatory CAR Truncated CD19-expressing Autologous T-Lymphocytes
NCT03638206	Phase 1Phase 2	73 participants	1 March 2023	Number of participants with adverse events evaluated with NCI CTC AE, version 4.0 [Time Frame: 60 months]	Autologous CAR-T/TCR-T Cell Immunotherapy for Malignancies	CAR T-cell immunotherapy
NCT04003649	Phase 1	60 participants	31 December 2024	Incidence of adverse events [Time Frame: up to 15 years] DLT [Time Frame: up to 28 days] Feasibility (neoadjuvant therapy) [Time Frame: up to 14 days] Feasibility (adjuvant therapy) [Time Frame: up to 28 days] OS [Time Frame: at 9 months]	IL13Ra2-CAR T Cells with or without Nivolumab and Ipilimumab in Treating Patients with GBM	IL13Ralpha2-specific Hinge-optimized 4-1BB-co-stimulatory CAR/Truncated CD19-expressing Autologous TN/MEM Cells Ipilimumab Nivolumab
NCT04661384	Phase 1	30 participants	17 November 2025	Incidence of adverse events [Time Frame: up to 15 years] OS [Time Frame: at 3 months]	Brain Tumor-Specific Immune Cells (IL13Ralpha2-CAR T Cells) for the Treatment of Leptomeningeal Glioblastoma, Ependymoma, or Medulloblastoma	IL13Ralpha2-specific Hinge-optimized 41BB-co-stimulatory CAR Truncated CD19-Expressing Autologous T Lymphocytes
NCT05627323	Phase 1	42 participants	January 2041	DLT [Time Frame: 28 days] CRS [Time Frame: up to 15 years] All other adverse events and toxicities [Time Frame: up to 15 years]	A Phase 1 Study to Evaluate CHM-1101 CAR T Cells in Patients with MMP2+ Recurrent or Progressive Glioblastoma	CHM-1101 CAR T cells
NCT05577091	Phase 1	10 participants	1 November 2032	Safety: any adverse events associated with one or multiple autologous Tris-CAR T-cell infusions will be assessed by CTCAE v5.0. [Time Frame: 6 months]	Tris-CAR-T Cell Therapy for Recurrent Glioblastoma	Inverse correlated dual-target, truncated IL7Ra modified CAR -expressing autologous T lymphocytes
NCT05353530	Phase 1	18 participants	December 2040	Safety of 8R-70CAR T-cell therapy in adult patients with *de novo* CD70+ GBM [Time Frame: 28 days post-infusion] Feasibility of 8R-70CAR T-cell therapy in adult patients with *de novo* CD70+ GBM [Time Frame: 10 weeks]	Phase I Study of IL-8 Receptor-Modified CD70 CAR T-Cell Therapy in CD70+ and MGMT-Unmethylated Adult Glioblastoma (IMPACT)	*Ex vivo* expanded autologous IL-8 receptor (CXCR2) modified CD70 CAR (8R-70CAR) T cells
NCT04717999	Not Applicable	20 participants	31 December 2023	Number of participants who experience a DLT [Time Frame: 2 years]	Pilot Study of NKG2D CAR-T in Treating Patients with Recurrent Glioblastoma	NKG2D CAR-T
NCT05168423	Phase 1	18 participants	19 December 2039	Number of subjects with treatment-related adverse events using NCI CTC AE V5.0 [Time Frame: 15 years] Number of subjects with DLT [Time Frame: 12 months] Determination of maximum tolerated dose assessed by collection of adverse events as graded by CTCAE. [Time Frame: 12 months]	CART-EGFR-IL13Ra2 in EGFR Amplified Recurrent GBM	5×10^7^ CART-EGFR-IL13Ra2 1×10^7^ CART-EGFR-IL13Ra2 1×10^8^ CART-EGFR-IL13Ra2 5×10^8^ CART-EGFR-IL13Ra2 Cyclophosphamide Fludarabine

CAR T cell, chimeric antigen receptor T cell; DLT, dose-limiting toxicity; PFS, progression-free survival; MTD, maximum tolerance dose; RP2D, recommended phase 2 dose; OS, overall survival; PPS, post-progression survival; RD, recommended dose; NCI, National Cancer Institute; CTCAE, common toxicity criteria for adverse events; CRS, cytokine release syndrome.

Vaccines against glioblastoma are classified into five types: (1) dendritic cell vaccines, (2) polypeptide vaccines, (3) autologous vaccine, (4) heat-shock protein vaccines, and (5) viral protein-based vaccine (30). Immune checkpoints play a significant role in regulating immune response; thus, researchers have developed a series of immune checkpoint inhibitors (ICIs). ICIs can rejuvenate the immune system of children, preventing tumor growth and metastasis. The discovery of ICIs has been acknowledged as a major breakthrough in cancer treatment (31). The key ICIs targeted for glioma include the following: cytotoxic T- lymphocyte-associated protein type 4 (CTLA-4) and programmed cell death-1 (PD-1)/programmed cell death ligand-1 (PD-L1). These ICI s have been principally targeted to treat severe neoplastic diseases (32) and have achieved satisfactory results in some cancers. However, at present, ICI therapy still has serious side effects (e.g., drug resistance). Such side effects are usually related to the increase in immune system activity, which can be manifested as fatigue, anemia, neutropenia, hypothyroidism, rash, colitis, pneumonia and even myocarditis, hepatitis, and other serious adverse events. In terms of drug resistance, most patients show primary drug resistance, and some patients show acquired drug resistance after treatment reaction (31). Therefore, for the successful application of ICI treatment, one needs a comprehensive understanding of its toxicity and the development of new immune checkpoint molecules and combination with other treatments to overcome drug resistance.

PD-L1 expression in glioblastoma remains contradictory. Nonetheless, glioblastoma patients with a higher PD-L1 expression showed an impaired prognosis likely governed by immunosuppressive mechanisms. Nivolumab, which targets PD-1, did not improve longevity in patients diagnosed with recurrent glioblastoma. Alternatively, bevacizumab showed promising results in its phase III trial against glioma development (33). Collectively, immunotherapy for glioma demands further advanced clinical trials using a higher combination therapy that engages immunotherapies targeting the immune system and cells for the effective checkpoint suppression of cancer cells. We firmly believe that combined immunotherapy could have a major breakthrough in managing glioma.

## Hepatoblastoma

Hepatoblastoma is a highly prevalent liver malignancy among children accounting for 50%–60% of cases. In comparison, solid malignant tumor incidence accounts for 0.8%–2.0% in children. Hepatoblastoma akin to neuroblastoma and nephroblastoma has been identified as an intraperitoneal malignancy (34) that inflicts severe health ailments in early childhood. Hepatoblastoma, also classified as an embryonic tumor with its detrimental effects, remains unexplored. Clinicians presume that the onset of its adverse symptoms could occur during the advanced fetal stage or later during adulthood, as well as during the infant stage and early childhood. Children in the age group <3 years/<15 years are highly susceptible to the detrimental effects of hepatoblastoma compared to adults (35).

Children with hepatoblastoma showed adverse symptoms such as asymptomatic abdominal mass. Furthermore, other symptoms like fever, decreased body mass, anorexia, and obstructive jaundice are seldom observed among children with hepatoblastoma. Clinical therapies recommended for treating hepatoblastoma include surgical procedures, radiotherapy, liver transplantation, and chemotherapy (36). A mong these, immunotherapy, which principally engages active immune responses, is exceptional in managing hepatoblastoma. Immunotherapy possessed explicit clinical merits on children with hepatoblastoma demanded prolonged radiotherapy and chemotherapy. Clinical pathways and peptides linked to hepatoblastoma have been explored meticulously. Immunosuppressants related to these therapeutic targets were effective in dealing with refractory hepatoblastoma on child patients found immune to surgical procedures, radiotherapy, and chemotherapy.

**Figure 5:**
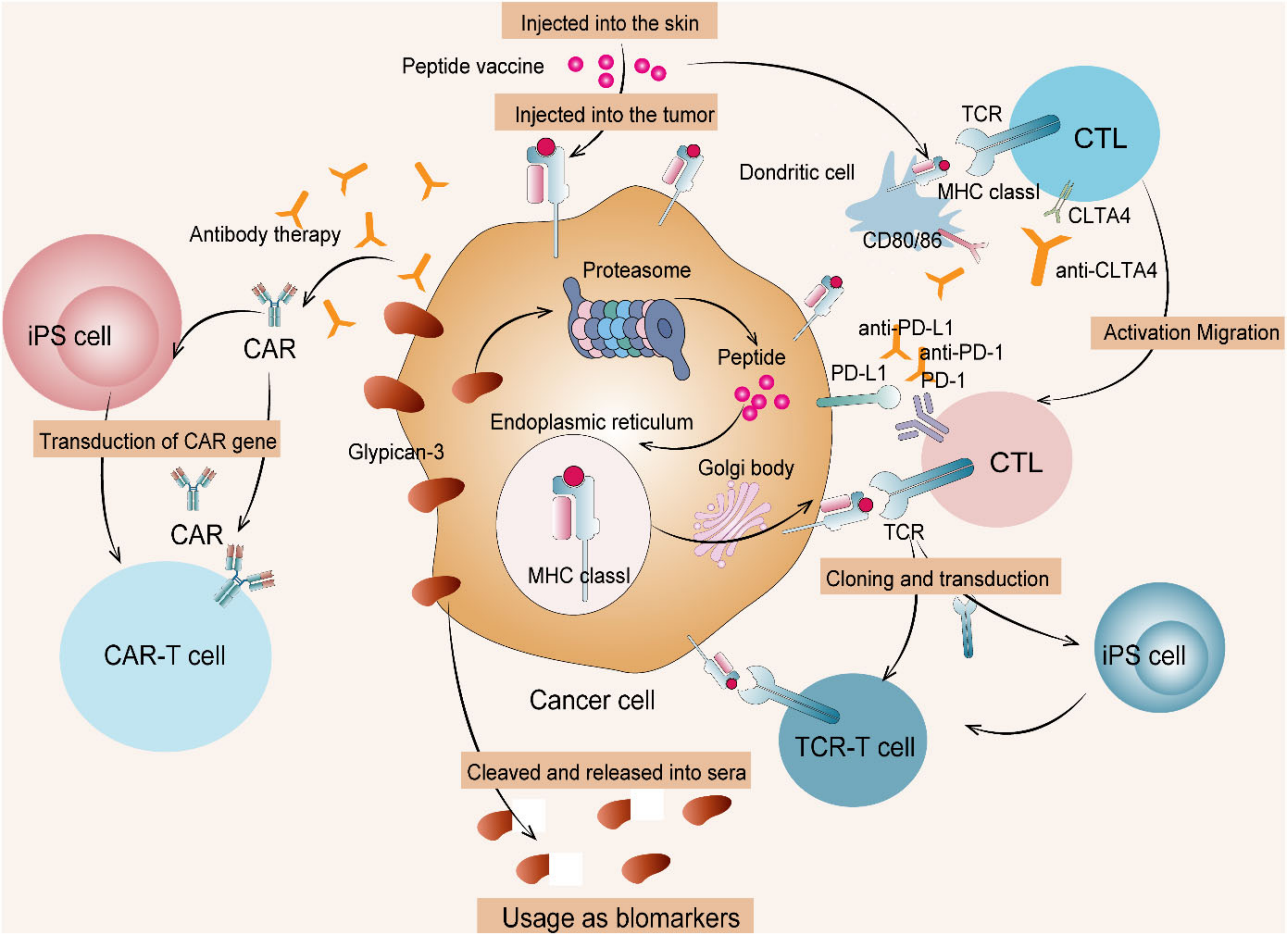
Cancer immunotherapy targeting GPC3. (1) Therapies targeting intracellular GPC3 mainly include GPC3 peptide vaccine and adaptive immunotherapy based on T cells transduced with a suitable TCR. (2) Therapies targeting membrane-bound GPC3 mainly include antibody therapy and anti-GPC3-CAR T-cell therapy. (3) Such T cells and CAR T cells from induced pluripotent stem (iPS) cells are currently being developed. CTL, cytotoxic T lymphocyte; TCR, T-cell receptor.

Common inhibitory protein targets of hepatoblastoma are associated with classical tumor pathways and peptides, particularly glypican-3 (GPC3). GPC3, which is a 65- kDa membranous receptor protein with 580 amino acids, is identified as one of the heparan sulfate proteoglycans (37). GPC3 is ubiquitously expressed on tumor cell membranes, including hepatocellular carcinoma, ovarian clear cell carcinoma, melanoma, lung squamous cell carcinoma, hepatoblastoma, Wilms tumor, and yolk sac tumor. Moreover, GPC3 critically regulates the classical Wnt/β-catenin pathway activation, which is a notable therapeutic means of managing the above deleterious tumor conditions, especially hepatoblastoma (38). GPC3 is also a notable therapeutic target in immunotherapy used for attenuating solid tumor growth in younger children. Immunosuppressants coupled with other immunotherapies, such as tumor vaccine, monoclonal antibody, antibody–drug conjugate, bispecific antibody, CTL, and CAR T-cell therapy specific to GPC3, could attenuate solid embryonic tumor in children (39). Peptide vaccines raised against tumor antigens have proven to be moderately effective in controlling solid refractory tumors in children (40). The GPC3 peptide vaccine exclusive to GPC3-+ve hepatoblastoma has remarkably controlled its emergence at the second level in affected children. Concordantly, a monoclonal antibody raised against GPC3 tumor antigen attenuated hepatocellular carcinoma in patients enrolled in a clinical phase I trial study (41). It is more likely for this GPC3-specific monoclonal antibody to exhibit a similar anti-tumor effect in children with hepatoblastoma. Noticeably, children diagnosed with hepatoblastoma aged >1 year responded well to the GPC3 peptide vaccine than the lower age group children (42). The study defines the age limit for children with hepatoblastoma who intend to undertake immunotherapy, especially the GPC3 peptide vaccine. The mechanism of cancer immunotherapy targeting GPC3 is shown in **Figure 5**. There are only two clinical trials in progress of GCP3 for hepatoblastoma now, as detailed in **Table 4**.

**Table 4:** Clinical trials in progress of GCP3 for hepatoblastoma.

ClinicalTrials.gov Identifier	Type of study	The number of estimated enrollment	Estimated study completion date	Current primary outcome measures	Brief title	Drugs and methods
NCT04093648	Phase 1	42 participants	January 2038	DLT Rate [Time Frame: Start of lymphodepleting chemotherapy to 4 weeks post T-cell infusion] DLT rate will be calculated as the number of patients experiencing a DLT divided by the total number of patients receiving treatment. The NCI Common Toxicity Criteria V5.X will be used in grading toxicity with the exception of CRS and neurological toxicities that are related to T-cell infusions. CRS and neurological toxicities will be graded according to Appendix VI in the protocol. Response rate according [Time Frame: 8 weeks post T-cell infusion] Response rate will be estimated as the percentage of patients whose best response is either complete remission or partial remission using the international criteria proposed by the RECIST Committee and by the Immune-related Response Criteria.	T Cells Co- Expressing a Second-Generation Glypican 3-Specific Chimeric Antigen Receptor with Cytokines Interleukin-21 and 15 as Immunotherapy for Patients with Liver Cancer (TEGAR)	Genetic: TEGAR T cells Drug: Cytoxan Drug: Fludarabine
NCT04928677	Phase 1	20–489 participants	June 2024	(1) Estimate the MTD [Time Frame: 1 year] The Phase A dose escalation scheme will follow a 3 + 3 design. (2) Response rate [Time Frame: at 6 weeks] partial + complete response according to RECIST v1.1)	A Study of Codrituzumab in Children and Young Adults with Solid Tumors and Have Not Responded to Treatment or Have Come Back After Treatment	Codrituzumab

DLT, dose-limiting toxicity; RECIST, Response Evaluation Criteria in Solid Tumors.

## Pulmonary blastoma

Pulmonary blastoma, defined as a sarcomatoid carcinoma of the lung, is rarely seen among children and accounts for <0.5% of cases. According to the article of Koss, pulmonary blastoma has been bifurcated into three major subtypes: (1) classic biphasic pulmonary blastoma (CBPB), (2) pleuropneumoblastoma, and (3) adequately differentiated fetal adenocarcinoma (43). Pleuropulmonary blastoma is a highly prevalent preliminary lung malignant tumor diagnosed in children (44).

The histopathological examination of pleuropulmonary blastoma tissue morphology showed epithelium and stroma with unevenly branched glandular structures. Additionally, hemorrhage and necrosis were seen on the tumor tissue cells. It is likely that children diagnosed with pleuropulmonary blastoma in their early stages were merely subjected to surgical therapy (45).

Lobectomy is the widely used surgical methodology to treat pleuropulmonary blastoma (46). Chemotherapy and radiotherapy are other mainstream treatment methods recommended for pleuropulmonary blastoma management. There is a lack of research studies that illustrate the merits of target-based therapy of pleuropulmonary blastoma. A case report on a pleuropulmonary blastoma child with CD74-ROS1 gene mutation showed that the child positively responded to the chemo drug crizotinib (47). The case study warranted further in- depth clinical studies to confirm the correlation between this gene mutation and the pleuropulmonary blastoma incidence. Moreover, the case study also emphasized exploring more detrimental gene mutations linked to pleuropulmonary blastoma.

## Pancreatic blastoma

Pancreatic blastoma is a malignant tumor of indigenous cell origin seldom diagnosed in children. In compliance with the detrimental stages of pancreatic blastoma emergence, treatment procedures such as surgical therapy, chemotherapy, and radiotherapy would be chosen. Surgical therapy remains the highly preferred therapeutic procedure for managing pancreatic hepatoblastoma among children wherein nearly 200 patients underwent surgical therapy worldwide (48). Further studies should be conducted to understand the therapeutic efficacy of immunotherapy against pancreatic hepatoblastoma.

## Nephroblastoma

Nephroblastoma is another common abdominal solid tumor and kidney tumor in children akin to neuroblastoma with a higher incidence (49). Surgical procedures augmented with radiotherapy and chemotherapy have proven to be effective, with a recovery rate of 90%. Nevertheless, this treatment method showed noticeable side effects with adverse toxicity on treated children with nephroblastoma (50).

Children < 10 years of age are highly susceptible to nephroblastoma. The age group between 2 and 5 years was identified to be crucial with a higher incidence of nephroblastoma (51). Chemotherapy drugs of recent use inflict higher mortality within its short interval of exposure governing other detrimental ailments of heart, lung, and kidney, including infertility and secondary tumor development eventually on the patients (52). It is likely that immunotherapy can exhibit a noticeable therapeutic effect but with trivial side effects on nephroblastoma patients. Macrophage infiltration is ubiquitous in all cancerous tissues observed at the highest level in all tumor states, including nephroblastoma.

A literature survey of 230 research studies on nephroblastoma revealed 23 studies on immunotherapy conducted since 2003, of which 17 studies reported in the past decade focused on immunotherapy. The notable immunotherapy procedures reported in the literature review included target-based immunotherapeutic drugs, peptide vaccines, and other cancerous cell surface components of the target (53–56).

CD3+ T lymphocytes expressed at a negligible level by nephroblastoma are likely to trigger immune responses presided by T-regulatory/helper cells (55, 56). Moreover, nephroblastoma tissue cells with elevated interleukin-10 (IL-10) and indoleamine 2,3 dioxygenase expression can inhibit host–immune responses with enhanced immunosuppressant action. Tumor-specific cytotoxic T-cell recruitment preventing nephroblastoma tumor metastasis is feasible by administering T-cell vaccine or CAR T-cell generation (53). Clinical trials in progress of CAR-T treatment for nephroblastoma are detailed in **Table 5**.

**Table 5:** Clinical trials in progress of CAR-T treatment for nephroblastoma.

ClinicalTrials.gov Identifier	Type of study	The number of estimated enrollment	Estimated study completion date	Current primary outcome measures	Brief title	Drugs and methods
NCT04377932	Phase 1	24 participants	1 February 2040	Number of patients with DLT [Time Frame: 4 weeks]	Interleukin-15 Armored Glypican 3-Specific Chimeric Antigen Receptor Expressed in T Cells for Pediatric Solid Tumors	AGAR T cells (GPC3-CAR and the IL15) Cytoxan Fludara
NCT04715191	Phase 1	24 participants	3 July 2041	Number of patients with DLT [Time Frame: 4 weeks]	Interleukin-15 and -21 Armored Glypican-3-Specific Chimeric Antigen Receptor Expressed in T Cells for Pediatric Solid Tumors	CARE T cells (GPC3-CAR and the IL15 plus IL21) Cytoxan Fludara
NCT04897321	Phase 1	32 participants	1 March 2027	Safety of B7-H3-CAR T cells [Time Frame: 6 weeks after B7-H3-CAR T-cell infusion]	B7-H3-Specific Chimeric Antigen Receptor Autologous T-Cell Therapy for Pediatric Patients with Solid Tumors (3CAR)	Fludara, Cytoxan, MESNA, B7-H3 CAR T cells
NCT03618381	Phase 1	36 participants	June 2038	MTD and DLT, and describe the full toxicity profile of the two CAR T-cell products [Time Frame: 28 days]. The number of successfully manufactured EGFR806 and EGFR806xCD19 CAR T cell products will be assessed [Time Frame: 28 Days]. Establish the safety, defined by adverse events, of EGFR806-specific CAR T-cell infusion s (Arm A), and of dual transduced EGFR806xCD19 CAR T-cell infusion s (Arm B) [Time Frame: 28 Days]	EGFR806 CAR T-Cell Immunotherapy for Recurrent/Refractory Solid Tumors in Children and Young Adults	Second-generation 4-1BBζ EGFR806-EGFRt, second -generation 4-1BBζ EGFR806-EGFRt, and a second-generation 4 1BBζ CD19-Her2tG
NCT04483778	Phase 1	68 participants	December 2040	Assess the safety and tolerability of cellular immunotherapy utilizing *ex vivo* expanded autologous T cells genetically modified to express B7H3-specific CAR (Arm A) [Time Frame: 28 days] Assess the safety and tolerability of cellular immunotherapy utilizing *ex vivo* expanded autologous T cells genetically modified to express a bispecific B7H3xCD19 CAR (Arm B) [Time Frame: 28 days] Determine the MTD of B7H3-specific CAR (Arm A) [Time Frame: 28 days] Determine the MTD of bispecific B7H3xCD19 CAR (Arm B) [Time Frame: 28 days] Assess the DLTs and describe the full toxicity profile for each study arm [Time Frame: 28 days] Assess the feasibility of manufacturing B7H3 specific CARs from patient-derived lymphocytes [Time Frame: 28 days] Assess the feasibility of manufacturing B7H3xCD19 bispecific CARs from patient-derived lymphocytes [Time Frame: 28 days]	B7H3 CAR T-Cell Immunotherapy for Recurrent/Refractory Solid Tumors in Children and Young Adults	Second-generation 4-1BBζ B7H3-EGFRt-DHFR, second -generation 4-1BBζ B7H3-EGFRt-DHFR (selected), and a second-generation 4-1BBζ CD19-Her2tG

DLT, dose-limiting toxicity; RECIST, Response Evaluation Criteria in Solid Tumors. MTD, maximum tolerated dose.

The clinical phase I trial performed using tumor-centric antigen focusing on the recruitment of cytotoxic T cells inhibited the development of pediatric-solid tumors in relapsed/refractory states on the respective study patients (54). Biological components of tumor cells such as polysaccharides, glycoproteins, and cell surface receptors play vital roles in tumor cell metastasis governed by tumor cell- to-cell communication, proliferation, and infiltration. Target-based tumor therapy engages cell surface molecules such as GM3 and GPC3, which are promising and effective in treating nephroblastoma (40, 57, 58).

Collectively, research studies that focused on immunotherapy against nephroblastoma included cell surface components, cytokines, and tumor -suppressing genes. These proposed research studies recommend immunotherapy as a treatment to manage nephroblastoma (59).

## Conclusion and future prospects

The prognosis of patients diagnosed with malignant blastoma was poor and disappointing, with recommended therapeutic treatment methods such as active surgery, chemotherapy, and radiotherapy. Patients subjected to chemotherapy and radiotherapy suffered from severe secondary health complications, eventually adversely affecting their lifestyle and livelihood. Recent ly, immunotherapy that employs monoclonal antibodies, cancer vaccines, and CAR cell therapy in managing malignant blastomas in affected patients shows promise and is effective (16, 60). Although a few novel therapeutic approaches excluding immunotherapy have been subjected to clinical trials, it is still time-consuming and challenging to implement strategies that are clinically based on study outcomes in treating malignant blastomas. We firmly believe that having immunotherapy as an adjunct therapy could benefit patients with malignant blastoma in terms of reduced mortality and increased life expectancy with sustainable clinical outcomes.

## Data availability statement

The original contributions presented in the study are included in the article/supplementary material. Further inquiries can be directed to the corresponding authors.

## Author contributions

BZ collected and analyzed data. LD and LL wrote the manuscript. SAK revised this manuscript. YD designed the study. WL and CZ collected and analyzed data and supervised the drafting of the manuscript. All authors contributed to the article and approved the submitted version.
